# Mucosal-associated invariant T cells in infectious diseases of respiratory system: recent advancements and applications

**DOI:** 10.1186/s12950-024-00376-z

**Published:** 2024-02-28

**Authors:** Xue Lin, Ye Wang, Yanqi He

**Affiliations:** Department of Pulmonary and Critical Care Medicine, West China Hospital, Sichuan University, Chengdu, China

**Keywords:** MAIT_1_, MR1_2_, 5-OP-RU_3_, Immune barrier_4_, Infectious diseases_5_

## Abstract

Mucosal-associated invariant T (MAIT) cells are an atypical subset of T lymphocytes, which have a highly conserved semi-constant αβ chain of T-cell receptor (TCR) and recognize microbe-derived vitamin B metabolites via major histocompatibility complex class I related-1 molecule (MR1). MAIT cells get activated mainly through unique TCR-dependent and TCR-independent pathways, and express multiple functional and phenotypic traits, including innate-like functionality, T helper (Th) 1 cell immunity, Th 17 cell immunity, and tissue homing. Given the functions, MAIT cells are extensively reported to play a key role in mucosal homeostasis and infectious diseases. In the current work, we review the basic characteristics of MAIT cells and their roles in mucosal homeostasis and development of respiratory infectious diseases as well as their potential therapeutic targets.

## Introduction

Mucosal-associated invariant T (MAIT) cells, an innate subset of T cell, express a semi-invariant T cell receptor (TCR), which is widely found in mucosal tissues and plays a vital role in the mucosal barrier. Due to its unique mode of activation and phenotype, MAIT cells demonstrate special antibacterial and immunomodulatory activities. With the recent research advancements in MAIT cells and development of novel activators, their roles in a variety of conditions are gradually revealed, especially infectious diseases.

## About MAIT cells

Porcelli et al*.* reported a class of T cells with a constant TCR chain as early as in 1993 [1] before Treiner et al. introduced the concept of MAIT cells in 2003, who found major histocompatibility complex class I related-1 molecule (MR1) to be the limiting structure for MAIT cell activation [2]. In 2012 Kjer-Nielsen et al*.* demonstrated that the metabolic products of vitamin B could act as ligands for MR1 and bind to MAIT cells. Interestingly, the metabolite of vitamin B_2_ can effectively activate MAIT cells via the TCR pathway when bound to MR1. In contrast, 6-formylteropterin (6-FP), the metabolite of vitamin B_9_, fails to activate MAIT cells although it binds to MR1 as well [3], an interesting and specific activation modality that set the stage for numerous subsequent studies. Classical MAIT cells are currently considered to be a class of lymphocytes with dual functions of innate and adaptive immunity, with a specific phenotype of TCR_Va_7.2^+^ CD3^+^ CD161^+^, which can be expressed as different subtypes depending on the activation status of the cells. In addition to classical MAIT cells, some TRAV1-2 negative cells can also function through MR1 activation, which can be classified as “classical MAIT, non-classical MAIT and MR1-T” according to surface markers and activation characteristics [4, 5], but there are fewer studies and the definition is not standardised, and their function still needs further study. However, there are few studies and the definitions are not standardised and their functions need further investigation. In this section, we focus on the number, distribution, and cellular phenotype of classical MAIT cells.

## Number and distribution

**Fig. 1:**
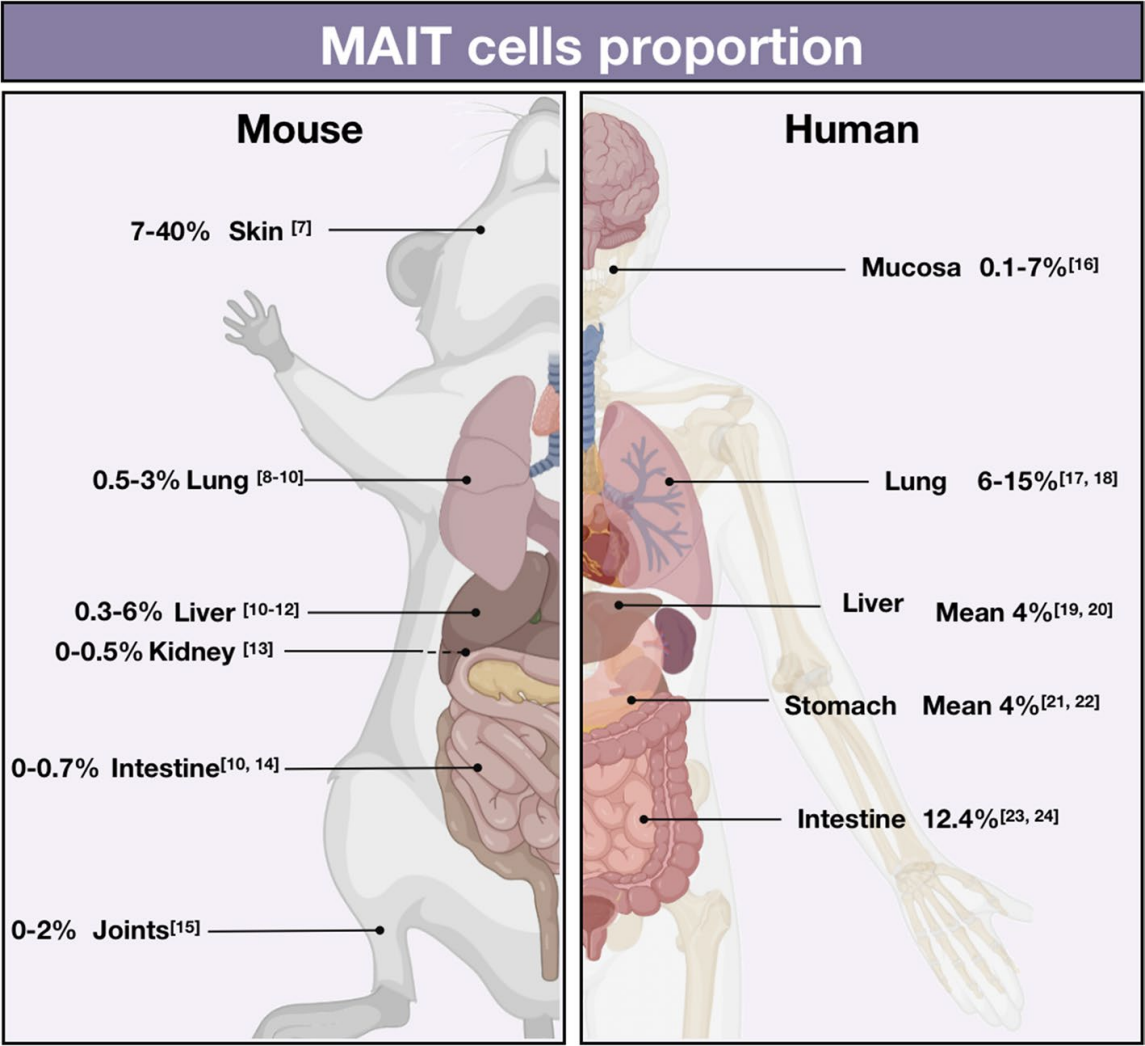
Frequency of MAIT cells (MAIT cells / αβ T cells, %) in mouse and human organs [7–24]

The sequence homology between the α1 and α2 structural domains of human and mouse MR1 is high at 90%. However, the number of MAIT cells in SPF experimental mice is relatively small -approximately 0.01% to 5% of αβ T cells [6]. This indicates a possible dependence of MAIT cell expansion on bacteria. Prevalence of MAIT cells in specific tissues and organs also varies due to factors such as activation mode and phenotype (Fig. 1).

## Phenotype

**Fig. 2:**
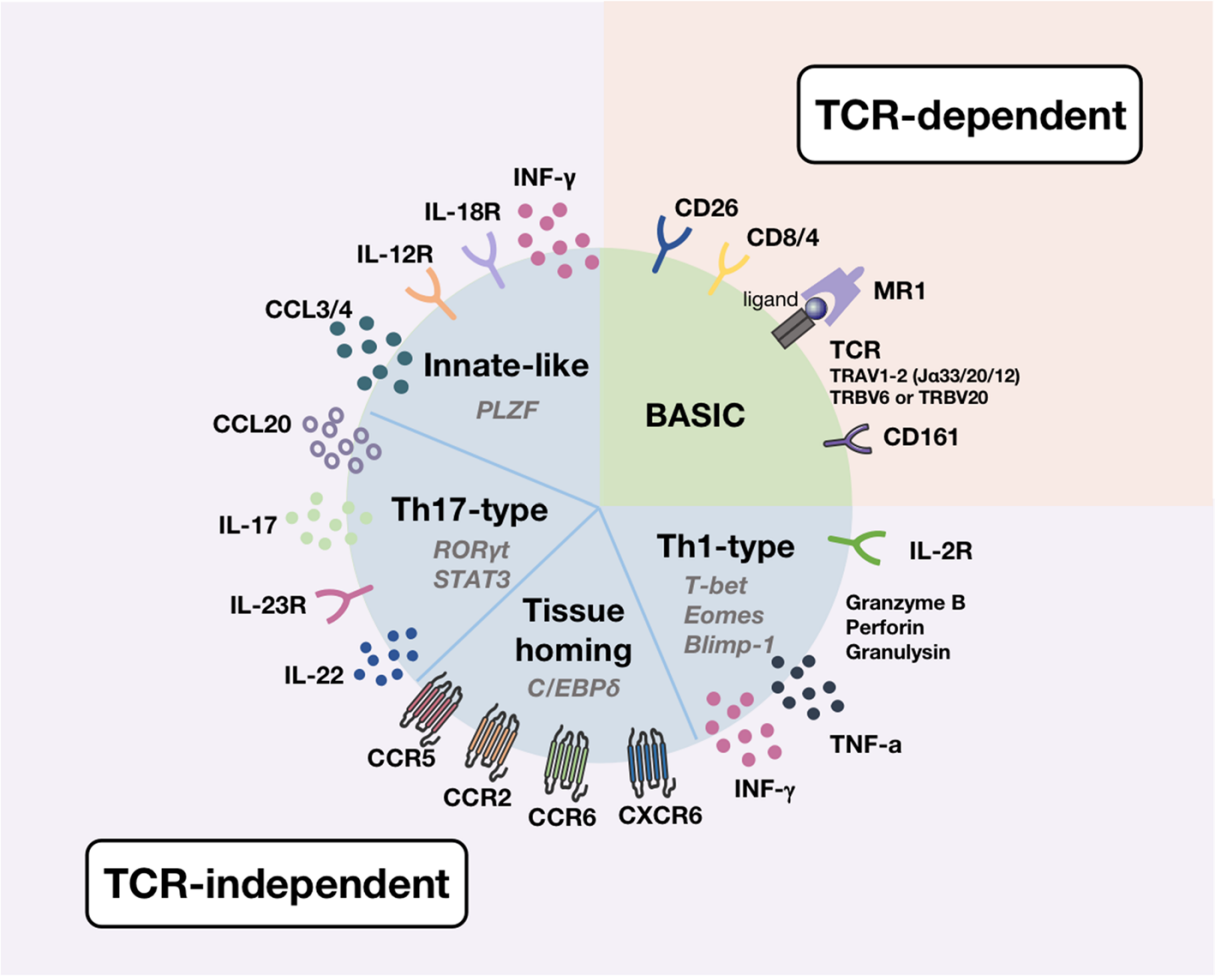
Phenotypic characteristics of MAIT cells. Note: Functionalities are as follows: The *BASIC* part of this figure shows the markers of the TCR-dependent functions; The other parts show the TCR-independent functions (Innate-like, Th1-type cell, Th17-type cell, tissue homing functions) via the experience phenotype genes

MAIT cells develop in the thymus, where immature thymocytes expressing the MAIT TCR interact with MR1 thymocytes expressing CD4^+^ CD8^+^ double positivity to trigger the intrathoracic developmental pathway [25]. MAIT cells are divided into three groups based on the expression of the phenotypic markers of CD4 and CD8: CD4^+^ cells, CD8^+^ cells, and CD8^−^ CD4^−^ double-negative cells [26]. The mature MAIT cells then enter the peripheral circulation and tissues to function accordingly. MAIT cells have a unique genetic rearrangement of the restricted TCR, which in humans is encoded by TRAV1-2 with the TCR α chain bound to TRAJ33, TRAJ12, or TRAJ20 and paired with a limited TCR β chain, usually encoded by TRBV6 or TRBV20. In mice TRAV1 is linked to TRAJ33 and paired with a TCR β consisting of a variable region of TRBV19 or TRBV13 [5, 27, 28].According to their phenotypic characteristics, the following functions of MAIT cells have been reported, including functions related to chemokines (C–C motif chemokine ligands: CCL-3, CCL-4) and chemokine receptors (C–C motif chemokine receptors: CCR-5, CCR6, CCR9; C-X-C motif chemokine receptor: CXCR-6), T helper (Th)-type 1 cells (Interferon-γ [IFN-γ]; tumor necrosis factor-a [TNF-α]; IL-2Rβ), Th17-type cells (IL-17, IL-22), and other cytokines [29–31] (Fig. 2).

## Activation pathways

MAIT cells are activated via two pathways. Similar to other T cells, MAIT cells can be activated through the TCR pathway. Ligands such as microorganisms may use the riboflavin biosynthesis pathway to activate MAIT cells in an MR1-dependent TCR pathway, a process in which co-stimulation of CD28, Toll-like receptors (TLR) enhances TCR-mediated activation of MAIT cells [32]. Recently reported noval ligands, such as 5-(2-oxoethylideneamino)-5-dribitylaminouracil (5-OE-RU) and 5-(2-oxopropylideneamino)-5-dribitylaminouracil (5-OP-RU), which demonstrated the most potent activating effects to date, have drawn extensive attention [33, 34]. And 5-OP-RU-loaded MR1 tetramers are also routinely used to identify MAIT cells by flow cytometry. In contrast, prolonged signaling in the normal organismal environment fails to induce proliferation or production of sequential cytokines different from conventional T cells. This regulation may be critical in preventing inappropriate MAIT cell activation. High expression of CD25 and CD69, and high or low expression of CD161 as well as secretion of Th1 cytokines such as IFN-γ and TNF-α, and Th17-type cytokines are often noted in MAIT cell activation. In addition to pro-inflammatory cytokines, MAIT cells secrete granzymes (GzmA, GzmB, and GzmK) and perforin to lysate infected cells [6, 29].

In addition to the TCR pathway where MAIT cells are rapidly activated by MR1-expressing antigen-presenting cells (APCs) combined with ligands formed by vitamin B metabolites, there is a non-TCR-dependent pathway. Increasing evidence has indicated that these two activation pathways combined may play an important role in optimizing the functionality of MAIT cells [35, 36].

## Roles in maintaining immune barrier

Microbial communities may colonize the skin, oral cavity, respiratory tract, digestive tract, and genitourinary tract, and interact with, for example, immune cells on the mucosal surface to maintain homeostasis at the mucosal barrier [37, 38]. Growing evidence has shown that MAIT cells, which are widely present at mucosal sites, are key to protecting the mucosa from external microbial threats and maintaining the immune barrier [39]. In this section, we will discuss the functions of MAIT cells in protecting and maintaining the integrity of the mucosal barrier by reviewing their multifaceted roles and interactions with other mucosal cells. In this section, we will further clarify the function of MAIT cells in the protection and maintenance of the mucosal barrier by their functions and their interactions with other mucosal cells.

## Autonomous function

**Fig. 3:**
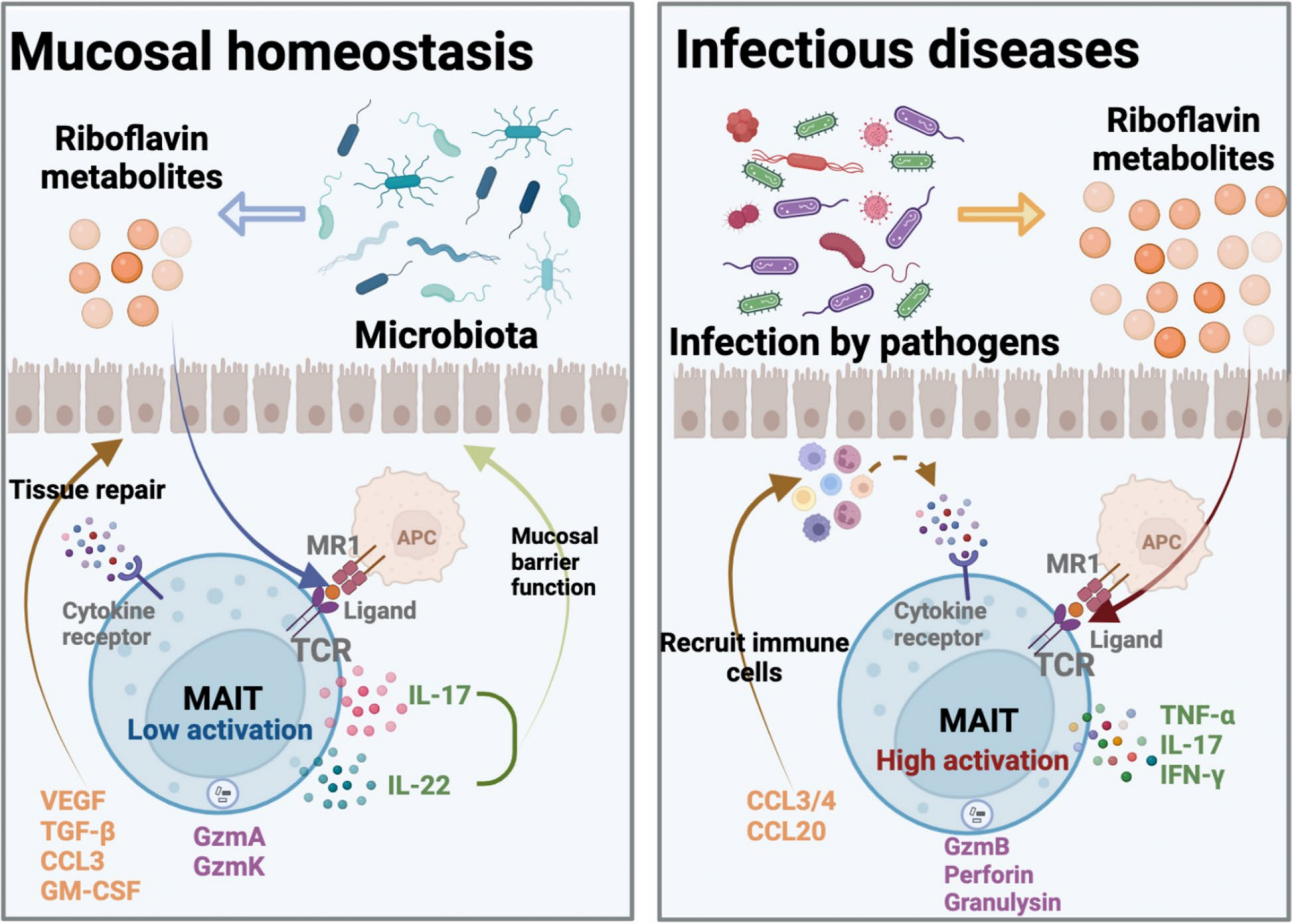
States and functions of MAIT in mucosal barrier homeostasis and infection

Microbial flora play a vital role in the maintenance of the mucosal immune barrier. Normal flora are in a dynamic balance in terms of species and numbers, interacting with host immune system to maintain homeostasis at the mucosal barrier, in which state MAIT cells have low cytotoxicity and release mostly GzmA and GzmK while GzmB and perforin are expressed at lower levels [40, 41]. MAIT cells can also show basal activity characterized by TCR-dependent activation by producing such cytokines as IL-17 and IL-22 to promote tissue repair and mucosal barrier function [42, 43]. Additionally, they may promote the expression of VEGF, TGF-β, CCL3, and GM-CSF at their mucosal barriers via both TCR-dependent and non-TCR-dependent pathways for tissue repair [44, 45]. Both approaches may be essential for maintaining mucosal barrier functionality (Fig. 3, left).

In contrast, at the onset of infection, significant increases in the frequency of MAIT cells are seen at the site of infection whereas the number of MAIT cells decreases significantly in the peripheral blood [46]. This observation may be closely related to the type of pathogen, where the alteration in the number and function of MAIT cells are more pronounced when the pathogen contains enzymes related to riboflavin metabolism [47]. In addition to the quantitative changes, MAIT cells exhibited a significant elevation in their cytotoxicity, primarily releasing GzmB with a greater killing capacity. They also release TNF-α, IL-17, and IFN-γ to inhibit pathogen-associated functions. MAIT cells can secrete chemokines, which enables them to recruit other immune cells to the infection site rapidly after activation. This facilitates the formation of an interaction network between cells to play their parts at the mucosal barrier (Fig. 3, right). MAIT cells can exhibit diverse manifestations at different stages of infection. For instance, during the early stage of infection onset,they demonstrate an early cytotoxic response characterized by high expression of Forkhead box protein 3 (FOXP3) and GzmB. As the infection prolongs, they may display more heterogeneous phenotypic subpopulations, including proliferation, late activation, mast cell degranulation, and even depleted phenotypes [48].

Similar to other immunizations, excessive MAIT cell activation in the organism can lead to the development of local inflammatory storms, further damaging tissues at the site of the lesion. Interestingly, MAIT cells demonstrate the ability to self-adjust. While they typically exhibit pro-inflammatory features in most cases, an increasing number of studies have revealed potential immunosuppressive functions of MAIT cells. Several studies indicate that MAIT cells can exert immunosuppressive effects through IL-10, TGF-β, etc. [49, 50], suggesting a possible role of MAIT cells in the development of infection. The transition between pro- and anti-inflammatory states suggest that MAIT cells may have an essential function in the process of infection by adjusting their phenotype. How to exploit this mechanism to develop the function of MAIT cells in the prevention and treatment of infectious diseases are key to current research.

## Interaction with other immune cells in mucosal barrier

As mentioned above, the TCR activation pathway relies on APCs expressing MR1 receptors, such as dendritic cells, macrophages, and monocytes. These APCs activate MAIT cells by binding to riboflavin products produced by bacterial metabolism. The types of APCs responsible for MAIT cell activation vary between pathogens, potentially related to differences in cytokine expression and immune cells at the onset of infectious diseases.

For instance, at the site of infection, various cytokines, such as IL-7, IL-12, IL-15, and IL-18, directly activate MAIT cells [32, 51, 52]. IL-23, on the other hand, stimulates the accumulation of MAIT cells at the site of inflammation [53], and IL-2 further promotes the activation and leads to increased release of IFN-γ and GzB [54]. This implies that the degree of MAIT cell activation might be influenced not only by the pathogen but also by the status of other immune cells and cytokines [55–58].

In addition to activation by professional APCs, non-professional APCs can also activate MAIT cells in a TCR-dependent manner in certain diseases. For instance, during tuberculosis and typhoid infections, lung epithelial cells express MR1, which directly activate MAIT cells through a TCR-dependent pathway [17, 59].

The evidence presented above underscores the significance of MAIT cell interactions with other immune cells in mucosal tissue, which plays a key role in mucosal barrier homeostasis and the development of infections. Gaining a deeper understanding of the reciprocal immune relationships between MAIT cells, other cells, and cytokines within the mucosal barrier is crucial for further exploring the function of MAIT cells.

## Roles in infectious diseases of respiratory system

Due to their unique characteristics, MAIT cells hold a crucial function in preserving the integrity of the mucosal barrier in various tissues and organs, both in healthy states and disease processes [60]. These cells exert their functions through self-activation and interaction with other immune cells. And the microbiota in the normal respiratory system work together with immune cells, etc. to build up an immune balance [61, 62] to which MAIT cells can respond when this balance is disrupted by infection. Notably, lung MAIT cells secrete the directly antimicrobial molecule IL-26 and selectively express cytokine and chemokine-related molecules, such as IFN-γ and IL-12R [63]. These attributes further support MAIT cells as an early sensor in defending the respiratory barrier.

The activation of MAIT cells during bacterial infection primarily involves TCR-dependent and TCR-independent pathways. When antigen-presenting cells engulf and process pathogenic bacteria, the antigen binds to MR1, triggering the activation of MAIT cells in a TCR-dependent manner. This rapidly stimulates the releases of gamma interferon, TNF-a, and cytotoxic factors.

It is essential to note that not all infected cells can be activated through the non-TCR-dependent pathway. In some cases of infectious diseases, certain pathogenic bacteria may not activate MAIT cells to perform their physiological functions. However, the activation of MAIT cells is strongly correlated with the increased production of riboflavin intermediates in microorganisms. It has been reported that bacteria with riboflavin metabolic pathways can activate MAIT cells more effectively [47].

Interestingly, the immune microenvironment at the site of infection often exhibits variations at the onset of different infections. As a result, MAIT cells frequently demonstrate different activation states depending on factors such as the type of pathogen infection and the severity of the disease.. This diversity in activation states may suggest that MAIT cells do not uniformly perform the same function in different infectious diseases or at different stages of the disease. Notably, viruses do not synthesize riboflavin, and as a result, the regulation of MAIT cell function in viral infections is believed to be achieved through a TCR-independent mechanism [64, 65].

This section offers an overview of the significance of MAIT cells in respiratory diseases linked to bacterial, fungal, and viral infections.

### Bacterial infections

#### Mycobacterium tuberculosis

The role of MAIT cells in Mycobacterium tuberculosis (MTB) infection has been extensively studied in the last decade. MTB has been shown to specifically activate MAIT cells via the TCR pathway, and the overexpression of genes in the riboflavin-biosynthesis pathway has been found to attenuate the virulence of MTB [66]. It is widely recognized that MTB infection can activate MAIT cells not only through the TCR pathway but also the non-TCR pathways. Several studies [67, 68] have demonstrated that the proportion and absolute number of MAIT cells in the peripheral blood of patients with MTB are significantly lower than that of healthy volunteers. However, MAIT cells exhibit an increased capacity to release IFN-γ and GzB. The number of MAIT cells is significantly higher at the site of infection and in the respiratory tract. This correlates with their chemotactic ability to rapidly reach the site of infection and recruit other immune cells to perform their functions jointly. Interestingly, the glycolytic capacity of stimulated MAIT cells is significantly enhanced, possibly as an adaptive feedback to the low glucose levels in the diseased tissues [69, 70].

Further studies have indicated that the activity of MAIT cells varies during the different stages of MTB, where inactive MTB MAIT cells are relatively stable which perform functions similar to tissue repair. In contrast, upon the onset of active MTB infection, MAIT cells undergo prolonged activation, leading to a significantly increase in the number of PD-1-positive MAIT cells. This suggests that possible over-activation may further induce apoptosis of MAIT cells [68].

Based on the activation mode of MAIT cells, some scholars have proposed that specific activation of MAIT cells could be effective in preventing MTB infection or mitigating its severity. To this end, 5-OP-RU, a potent activation compound for MAIT cells, has been extensively studied in recent preclinical investigations.In one study, researchers utilized 5-OP-RU as an agonist to specifically activate MAIT cells after MTB exposure in mice. Surprisingly, the intranasal intervention with 5-OP-RU induced early activation and expansion of MAIT cells but did not attenuate the growth of MTB or the infection in the organism [71]. Similarly, another study exploring the preventive effect of 5-OP-RU vaccination after MTB infection found that MAIT cells failed to prevent MTB infection, and the intervention even delayed the initiation of specific CD4 + T cells after MTB infection. Consequently, this affected network interactions between MAIT cells and other cells, impacting further activation of MAIT cells [72].

Since the number, phenotype, and function of MAIT cells are different between mice and humans, some studies were conducted in rhesus monkeys. After 5-OP-RU treatment, MAIT cells did not expand but upregulated the expression of PD-1 which reduced their release of granzymes, cytokines, and other functions [73]. Several outcomes from this study indicate that treatment with 5-OP-RU does not improve infection control. Also noteworthy is the possibility that the application of 5-OP-RU for nasal dip induction may lead to bronchoconstriction, which complicates its therapeutic potential in the course of treatment.

In conclusion, existing preventive interventions may not be fully effective in preventing MTB infection. Further research is needed to develop and explore more targeted and efficient interventions that address the underlying mechanisms.

#### Legionella pneumophila

Legionella pneumophila is a parthenogenic intracellular pathogen known to cause Legionella pneumonia and Pontiac fever [74]. Notably, Legionella pneumophila possesses an intact riboflavin metabolic pathway that effectively activates MAIT cells. During the onset of infection, MAIT cells rapidly accumulate and activate at the site of infection during the onset of infection, displaying a cytotoxic function and exerting immune protective effects. This response is dependent on MR1, IFN-γ, and GM-CSF, while not relying on IL-17A, TNF, or perforin [8, 75].

Studies have explored the application of 5-OP-RU to activate MAIT cells, followed by re-inoculation with Legionella, which led to a significantly reducion in bacterial load and infection in the mouse organism. While IL-23 plus 5-OP-RU vaccine enhanced MAIT cell-mediated control of pulmonary Legionella infection and these findings provide evidence for the development of MAIT cells against infectious diseases [53], such findings need to be confirmed by further studies.

#### Other bacterial infections

Numerous studies have demonstrated that bacteria capable of metabolizing riboflavin can activate MAIT cells effectively, such as Escherichia coli, and Staphylococcus aureus [39]. Notably, Pseudomonas aeruginosa infection leads to a significant decrease in the number of MAIT cells in the peripheral blood, where the severity of the lung infection is negatively correlated with the number of MAIT cells [76].

In patients with community-acquired pneumonia, MAIT cell counts in peripheral blood have shown a correlation with disease severity. During antibiotic treatment and recovery from symptomatic remission, MAIT cell counts gradually recover [77]. Additionally, studies on sputum samples have revealded interesting findings. Rachel et al. [78] observed a higherabundance of MAIT cells in sputum in patients with mild community acquired pneumonia (CAP) compared to healthy controls. This abundance was associated with the presence of IFN-α, IFN-γ, and sputum neutrophil abundance. In Bronchoalveolar lavage fluid (BALF), MAIT cells expressing PLZF were significantly more prevalent in CAP patients, and their ability to secrete IL-17 was notably higher, suggesting possible functions at the site of infection [79].

Regarding Streptococcus pneumoniae infection, research has shown that the number of MAIT cells at the mucosa significantly increased after infection [80]. Nevertheless, despite these promising findings, more comprehensive basic and clinical evidence is still required to fully understand and support how MAIT cells can be effectively integrated into the prevention or treatment of infectious diseases.

### Fungal infections

Notably, some common fungal infection pathogens also possess the riboflavin metabolic pathway, such as Candida albicans, Candida smoothie, and Aspergillus, which can effective activate MAIT cells [81, 82]. Nevertheless, it is essential to acknowledge that fungal infections exhibit distinct MR1-dependent and TCR β-chain deviations compared to bacterial infections [83].

For instance, in the case of Mucorales, the activation of MAIT cells by Trichoderma reesei also requires the presence of riboflavin metabolites and was dependent on TCR involvement. This activation results in increased expression of CD69 and CD107a, whereas intracellular expression of perforin and GzmA was reduced [84]. However, despite the significance of these findings, there is currently limited literature exploring the relevance of fungi to MAIT cells. The association of MAIT cells with commensal fungal colonization or pathogenic fungal infections remains understudied, and further investigations are warranted to elucidate their potential relevance.

### Viral infections

Respiratory diseases caused by viral infections encompass a wide range of illnesses, including influenza virus, cytomegalovirus infections, and novel coronavirus infections. This section focuses on organismal infections with novel coronaviruses as a representative example. During infection with novel coronaviruses, the number of MAIT cells in peripheral blood decreases significantly, while MAIT cells in the respiratory tract become significantly enriched [85–87]. As the body recovers, the number and activation status of MAIT cells in peripheral blood gradually return to normal [88].

However, it is worth noting that the two phenotypes of MAIT cells, CD69high and CXCR3low, have been associated with poor clinical outcomes in patients with novel neocoronary pneumonia [89]. Moreover, an imbalance in the IFN-a-IL-18 axis has been shown to induce alterations in MAIT cell function, leading to cytotoxicity [90]. Notably, the level of IL-18 activation in vivo is positively correlated with the severity of infection and mortality. Consequently, over-activation of MAIT cells may result in an imbalance between protective and pathological immune responses.

Given the critical role that MAIT cells play in novel coronavirus infections, several studies have investigated the expression and activation of MAIT cells in relation to the likelihood and severity of Coronavirus Disease 2019 (COVID-19) infection in various populations. A study examining gender differences in MAT cell profiles following COVID-19 infection revealed a negative correlation between the frequency of infected circulating MAIT cells and the severity of infection. Interestingly, infected women had significantly fewer circulating MAIT cells but a significantly higher proportion of MAIT cells in the respiratory tract, with the cells showing greater activation [91]. This suggests that the specific protective MAIT cell profile observed in women may be associated with the lower severity of their infection.

Pregnant women, as a special group, have been suggested several studies to be generally more susceptible to viral infections. However, evidence indicates that risk factors for severe COVID 19 during pregnancy are similar to those of the general population. One study found that pregnant patients exhibit a stronger inflammatory response (elevated C-reactive protein and IL-6) and increased activation and chemotaxis of MAIT cells compared to non-pregnant infected women of the same age [92]. This suggests that pregnant women may possess a distinct immune capacity to cope with the onset of infection.

COVID-19 is an ever-evolving situation, and the remarkable effector and modulatory functions of MAIT cells make them a subject of significant interest in the context of the disease [93]. The wealth of evidence supporting their key role in COVID-19 underscores the potential of MAIT cells as targets for disease prevention and treatment. However, effectively harnessing MAIT cell immunity against pathogens requires a delicate balance to avoid excessive, premature, or dysregulated MAIT cell activation, which may lead to detrimental effects and organismal damage. Thus, unraveling the precise mechanisms of MAIT cell activation and regulation becomes crucial for developing strategies that maximize their protective functions while minimizing potential adverse outcomes.

## Conclusions and future perspectives

The evidence presented above highlights the significant role of MAIT cells in the body's innate and adaptive immunity. In respiratory infectious diseases, MAIT cells primarily function by releasing pro-inflammatory factors and lysis. The development of MAIT cell activation ligands such as 5-OP-RU, has brought light to this work. Nevertheless, current research has yet to identify an effective method of activating MAIT cells for the prevention or treatment of diseases. Combined with the specific mode of activation of MAIT cells that it may be able to play a relevant role in the prevention of infectious diseases, and by improving the time point of administration (pre-infection administration) or screening for better concentration and mode of administration may provide an opportunity for the realisation of this conjecture. Moving forward, it is essential to explore the delicate balance between the maintaining the mucosal barrier and managing the pro-inflammatory, cytotoxic profile of MAIT cells. This balance is crucial for the development MAIT cells as potential targets for disease prevention and treatment. It is worth noting that during an infectious disease, the organism may experience changes in the metabolic function of multiple tissues and organs, for example, in combination with metabolism-related disorders during an infection, a complexity that creates difficulties in intervening in the development of the disease. And many current mechanistic studies are based on in vitro or clean mice models, where animals are not exposed to pathogenic stimuli prior to the experiments. This scenario differs from real-life human infections, and strategies aimed at expanding MAIT cells through vaccination may not be as effective in humans as they are in clean conditions. Therefore, further research is necessary to elucidate the efficiency and safety of MAIT cell vaccination.

## Data Availability

Not applicable.

## Abbreviations

MAIT: Mucosal-associated invariant T
TCR: T-cell receptor
MR1: Major histocompatibility complex class I related-1 molecule
IL: Interleukin
6-FP: 6-Formylteropterin
CCL: C-C motif chemokine ligand
CCR: C-C motif chemokine receptor
CXCR: C-X-C motif chemokine receptor
Th: T helper
IFN-γ: Interferon-γ
T-Bet: T-box transcription factor 21
EOMES: Eomesodermin
Blimp-1: PR/SET domain 1
RORγt: RAR related orphan receptor C
STAT3: Signal transducer and activator of transcription 3
BALF: Bronchoalveolar lavage fluid
PLZF: Zinc finger and BTB domain containing 16
CAP: Community acquired pneumonia
COVID-19: Coronavirus Disease 2019
C/EBPδ: CCAAT/Enhancer Binding Protein δ
TLR: Toll-like receptors
5-OE-RU: 5-(2-Oxoethylideneamino)-5-dribitylaminouracil
5-OP-RU: 5-(2-Oxopropylideneamino)-5-dribitylaminouracil
Gzm: Granzymes
APCs: Antigen-presenting cells
TNF-a: Tumour necrosis factor-a
VEGF: Vascular endothelial growth factor
TGF-β: Transforming growth factor-β
GM-CSF: Granulocyte-macrophage colony stimulating factor
FOXP3: Forkhead box protein 3
MTB: Mycobacterium tuberculosis
